# Substantia nigra and locus coeruleus microstructural abnormalities in isolated rapid eye movement sleep behaviour disorder and Parkinson’s disease

**DOI:** 10.1093/braincomms/fcaf023

**Published:** 2025-01-21

**Authors:** Jacopo Pasquini, Michael J Firbank, Laura Best, Victoria Foster, Charlotte Stewart, Vincenzo Silani, Rory Durcan, Gemma Roberts, George Petrides, Roberto Ceravolo, David J Brooks, Kirstie N Anderson, Nicola Pavese

**Affiliations:** Clinical Ageing Research Unit, Newcastle University, Newcastle upon Tyne NE4 5PL, UK; Department of Clinical and Experimental Medicine, University of Pisa, Pisa 56126, Italy; Institute of Translational and Clinical Research, Newcastle University, Newcastle upon Tyne NE4 5PL, UK; Regional Neurosciences Centre, Royal Victoria Hospital, Belfast BT12 6PA, UK; Clinical Ageing Research Unit, Newcastle University, Newcastle upon Tyne NE4 5PL, UK; Clinical Ageing Research Unit, Newcastle University, Newcastle upon Tyne NE4 5PL, UK; Department of Neurology and Laboratory of Neuroscience, Istituto Auxologico Italiano IRCCS, Milan 20149, Italy; Department of Pathophysiology and Transplantation, Dino Ferrari Center, Università degli Studi di Milano, Milan 20122, Italy; Department of Geriatric Medicine, Beaumont Hospital, Dublin D09 V2N0, Ireland; Nuclear Medicine Department, Newcastle Upon Tyne Hospitals NHS Foundation Trust, Newcastle upon Tyne NE1 4LP, UK; Nuclear Medicine Department, Newcastle Upon Tyne Hospitals NHS Foundation Trust, Newcastle upon Tyne NE1 4LP, UK; Department of Clinical and Experimental Medicine, University of Pisa, Pisa 56126, Italy; Neurodegenerative Diseases Center, Azienda Ospedaliero Universitaria Pisana, Pisa 56126, Italy; Institute of Translational and Clinical Research, Newcastle University, Newcastle upon Tyne NE4 5PL, UK; Department of Nuclear Medicine and PET Centre, Institute of Clinical Medicine Aarhus University, Aarhus 8200, Denmark; Regional Sleep Service, Newcastle upon Tyne NHS Hospitals NHS Foundation Trust, Newcastle upon Tyne NE1 4PL, UK; Clinical Ageing Research Unit, Newcastle University, Newcastle upon Tyne NE4 5PL, UK; Department of Nuclear Medicine and PET Centre, Institute of Clinical Medicine Aarhus University, Aarhus 8200, Denmark

**Keywords:** iRBD, substantia nigra, locus coeruleus, NODDI, neuromelanin

## Abstract

Substantia nigra (SN) and locus coeruleus (LC) are two catecholaminergic, neuromelanin-rich nuclei that are affected in Parkinson’s disease (PD) and may show neuroimaging abnormalities before the onset of motor manifestations. The simultaneous, multimodal investigation of their microstructural abnormalities may provide useful insights on the spatial diffusion and tissue characteristics of neurodegeneration, and this may in turn help develop markers for disease-modifying clinical trials. Therefore, through neuromelanin-sensitive and diffusion MRI, we aimed to investigate microstructural abnormalities in those nuclei in isolated REM sleep behaviour disorder (iRBD) and PD. Fourteen participants with polysomnography-confirmed iRBD, 18 with PD and 18 healthy controls were scanned with structural, neuromelanin-sensitive and neurite orientation dispersion and density imaging (NODDI) MRI. iRBD participants also underwent dopamine transporter imaging. SN neuromelanin and NODDI diffusion parameters and LC neuromelanin signals were extracted. Motor and global cognitive assessments were also collected. iRBD and PD participants showed significantly reduced neuromelanin contrast in the LC middle section compared with healthy controls. PD also showed significantly reduced caudal LC and posterior SN neuromelanin signal. No differences in SN NODDI parameters were detected between iRBD and healthy controls. Five iRBD participants showed reduced striatal dopamine transporter. In the combined disease groups (iRBD and PD), significant associations were shown between SN neuromelanin signal and neurite density index (*r* = −0.610, corr-*p* = 0.001) and between SN neurite density index and free water fraction (*r* = 0.417, corr-*p* = 0.042). In the same group, motor scores were negatively associated with nigral neuromelanin signal (*r* = −0.404, corr-*p* = 0.044) and free water fraction (*r* = 0.486, corr-*p* = 0.018). In conclusion, iRBD participants showed significant neuromelanin loss in the LC, with a minority showing initial nigrostriatal dopaminergic abnormalities. Across the entire iRBD–PD spectrum, the association between SN neuromelanin signal loss, diffusion parameters and motor scores has the potential to capture different yet related aspects of SN degeneration.

## Teaser

Pasquini *et al*. explore microstructural abnormalities in LC and SN through neuromelanin and diffusion MRI in Parkinson’s disease and iRBD. They also show associations across SN microstructural abnormalities and with motor impairment in this disease spectrum.

## Graphical Abstract

**Graphical Abstract:**
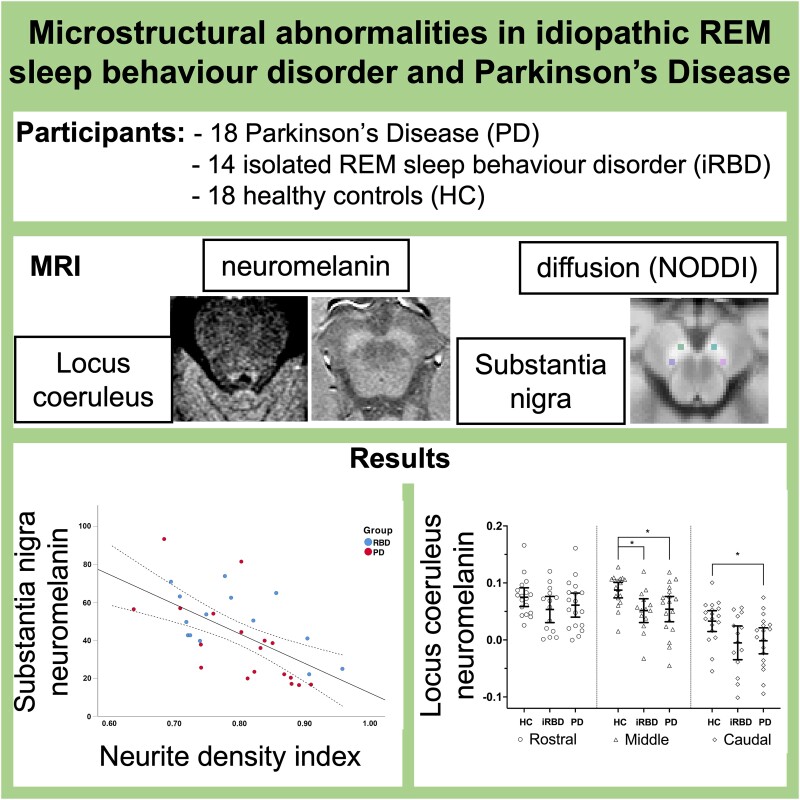

## Introduction

Isolated rapid eye movement (REM) sleep behaviour disorder (iRBD) is characterized by dream enactment and loss of normal muscle atonia^1^ and is a well-established premotor manifestation of Parkinson’s disease (PD) and other parkinsonian disorders with pathological alpha-synuclein.^2-5^

Substantia nigra (SN) pars compacta and locus coeruleus (LC) are two catecholaminergic, neuromelanin-rich nuclei where degeneration and neuronal loss start before the onset of the classic PD motor symptoms.^6-8^ Neuromelanin-sensitive MRI captures neuromelanin loss that parallels the degeneration of these nuclei in synucleinopathies, giving the possibility to track the neurodegenerative process *in vivo*.^9-14^ Alongside neuromelanin loss, SN tissue microstructural abnormalities may be captured by diffusion MRI (dMRI), a technique that exploits water motion to generate MRI contrast. Previous studies have employed diffusion tensor imaging (DTI) and free water-corrected, two-compartment model, to detect SN microstructural abnormalities. DTI findings have been inconsistent,^15,16^ but previous studies have reported an increase in SN free water in PD^17-21^ and iRBD,^22^ likely linked to tissue remodelling related to neurodegeneration.^23,24^

Recently, SN and LC neuromelanin-sensitive imaging and SN free water pools have been suggested as useful ‘disease-state’ biomarkers in both iRBD and PD.^16^ Furthermore, these techniques may be potentially useful as progression biomarkers in PD.^16^

While a few previous studies have used a combined dMRI and neuromelanin-sensitive MRI approach to investigate SN changes,^25,26^ studies that use these MRI techniques to simultaneously investigate SN and LC degeneration are lacking. The combined investigation of these nuclei in both premotor and motor stages of parkinsonism may provide useful insights on the extent and spatial distribution of brain microstructural abnormalities.^27,28^

In the current study, we used neuromelanin-sensitive MRI to investigate neuromelanin SN- and LC-associated signal and neurite orientation dispersion and density imaging (NODDI) to investigate dMRI tissue characteristics in the SN. NODDI is a multishell dMRI technique based on a three-compartment model that quantifies tissue free water pool along with other diffusion parameters [neurite density index (NDI) and orientation dispersion index (ODI)].^29,30^ These parameters capture different microstructural characteristics of brain tissue. Free water fraction (FWF) is the tissue isotropic fraction (i.e. the fraction of freely diffusing water in a given tissue unit); it is highest in the cerebrospinal fluid and increases in tissue affected by neuroinflammation and atrophy.^24,29,31^ NDI is calculated as the tissue fraction in which water molecules flow with restricted and preferential directionality and represents axons and dendrites. In normal tissue, it is higher in white matter where axons are packed in bundles and lower in grey matter where axons and dendrites are interspersed with other elements (somas, non-neuronal cells, etc.).^29^ ODI is an index of the orientation distribution of neurites: it is lower in white matter where axons have a preferential orientation and higher in grey matter where neurites have many different directionalities.^29^

The aims of the study were to simultaneously describe microstructural (i.e. neuromelanin signal and diffusion parameters) abnormalities in the SN and LC of iRBD and PD compared with healthy controls (HC); to investigate whether and how such microstructural abnormalities parameters, acquired from different MRI techniques, are associated; and to investigate their clinical correlates.

## Materials and methods

### Participants

Fifty-one participants were enrolled: 19 with PD, 14 with iRBD and 18 HC. One PD participant was subsequently excluded due to severe MRI motion artefacts.

All participants were required to be aged 45–80 years and were recruited between June 2019 and September 2021 from the Newcastle upon Tyne NHS Clinics for Research and Service in Themed Assessment (CRESTA) and the Regional Sleep Service at Newcastle Hospitals NHS Trust, Newcastle upon Tyne, UK.

PD diagnosis was made according to UK Brain Bank Criteria^32^ by a movement disorder specialist; iRBD participants had polysomnography-confirmed iRBD according to established criteria.^1^ Before inclusion, iRBD participants underwent a full clinical history and examination to exclude a neurological condition, and none had another significant sleep disorder such as obstructive sleep apnoea.

The exclusion criteria for all participants were as follows: a diagnosis of atypical Parkinsonism, cognitive impairment (MMSE < 24 at screening visit) or meeting DSM V criteria for major neurocognitive disorder. HC were required to show no clinical neurological dysfunction or MRI structural brain abnormalities. Furthermore, HC had no history of iRBD based on their medical history, interview with bed partners and REM Sleep Behaviour Disorder Questionnaire (RBDSQ).

Ethical approval was granted by the London-Surrey Research Ethics Committee (18/LO/2123). All participants involved in the study provided written informed consent according to the Declaration of Helsinki. We confirm that we have read the Journal’s position on issues involved in ethical publication and affirm that this work is consistent with those guidelines.

### Clinical assessments

All participants underwent clinical assessment and filled the RBDSQ on the day of the MRI scan. Both iRBD and PD patients underwent the Movement Disorders Society—Unified PD Rating Scale (MDS-UPDRS) Parts I, II and III, and Montreal Cognitive Assessment (MoCA) examinations. Medication was not withheld prior to the investigations performed in this study.

### MRI acquisition

Images were acquired on a 3T PET-MR System (Sign, GE Healthcare, Milwaukee, WI, USA).

A T_1_-weighted 3D MRI was acquired through a sagittal fast spoiled gradient recall (FSPGR) sequence with inversion time 400 ms, echo time (TE) 3 ms, repetition time (TR) 7 ms, flip angle 11°, voxel size 1 × 1 × 1 mm and parallel acceleration factor = 2.

#### Diffusion MRI

Multishell diffusion-weighted echo-planar imaging was performed with TR 5500 ms and TE 102 ms. Multishell diffusion weighting was achieved with *b*-values = 0 (*n* = 10), 300 (*n* = 8), 700 (*n* = 30) and 2000 s/mm^2^ (*n* = 60). Seventy-four slices of 2.2 mm were acquired, with a field of view of 220 × 220 mm and an acquisition matrix of 100 × 100. Parallel acceleration factor = 2 and multiband acceleration factor = 3 were used. One volume with *b*-value = 0 and reverse phase-encoding direction was collected.

#### Neuromelanin MRI

Two different neuromelanin-sensitive sequences were performed, based on previous studies indicating differential contrast for LC versus SN.^33^

An axial magnetization transfer prepared (MT) 2D gradient echo-based (GRE) sequence, as in Langley *et al*.,^34^ was performed with TR 500 ms, TE 4.2 ms, flip angle 50°, susceptibility frequency offset 1200 Hz with flip angle 300°. Two acquisitions were performed.

The second sequence was based on a fast spin-echo (FSE) T_1_-weighted sequence, as described by Sasaki *et al*.,^35^ with TR 600 ms, TE 12 ms, flip angle 111° and two echo trains. Three acquisitions were performed.

For both these sequences, 13 slices with a slice thickness of 2.5 mm, plus a 0.3-mm interslice gap, and a field of view of 220 × 165 mm were acquired with a 512 × 384 acquisition matrix.

Slices were acquired perpendicularly to the posterior aspect of fourth ventricle.

### MRI data processing

#### Diffusion MRI

dMRI data were pre-processed using PreQual (version 1.0.6)^36^ which is built around the MRTrix3,^37^ FSL^37^ and ANTs^38^ software. Data were denoised with MRTrix3 dwidenoise (MP-PCA) function. Images were then intensity-normalized to the first image and concatenated for further processing. FSL’s topup and eddy algorithms were used to correct for susceptibility-induced and motion artefacts and eddy currents and to remove outlier slices. Lastly, the pre-processed data were fitted with a tensor model using the FSL dtifit command. The quality control output was visually inspected for all subjects.

The Accelerated Microstructure Imaging via Convex Optimization (AMICO) package (https://github.com/daducci/AMICO) NODDI model was applied to pre-processed data.^39^

Before extracting dMRI parameters from the SN, a diffusion tensor-based analysis approach was used to register the diffusion tensor data from all participants to a study-specific template with DTI-TK software (http://dti-tk.sourceforge.net/pmwiki/pmwiki.php).^40^ The diffusion parameter images from NODDI and structural scans were transformed into the template space.

To ensure the correct placement of the four (two on each side) SN regions of interest (ROIs), an average neuromelanin template was created by registering (using SPM’s co-register) the neuromelanin MT-GRE scan of each participant with their *b* = 0 diffusion scan and then transforming the co-registered neuromelanin scan to the study-specific diffusion template space using the already calculated DTI-TK transforms. SN ROIs were placed rostro-medially [anterior SN (aSN)] and ventro-laterally [posterior SN (pSN)] on each side. ROIs covered (unilaterally) eight voxels each (Supplementary Fig. 1A–C), as in previous studies.^17,19^

Finally, mean values of NDI, ODI and FWF were extracted from each SN region.

#### Neuromelanin MRI

To estimate SN neuromelanin-associated signal, the two MT-GRE images of each participant were co-registered and averaged with SPM (https://www.fil.ion.ucl.ac.uk/spm/). Neuromelanin levels were estimated following Schwarz *et al*.^41^ This method estimates a hyperintense volume in the SN exceeding a predefined background threshold, corresponding to voxels containing neuromelanin. aSN, pSN and cerebral peduncles ROIs were manually drawn using ITK-SNAP (http://www.itksnap.org/)^42^ on three consecutive slices best displaying the typical SN hyperintensity. Using FSLUTILS in FSL, the cerebral peduncle’s mean intensity and SD were determined; then, the volume of SN voxels exceeding mean + 3.25 SD intensity was calculated for aSN and pSN bilaterally. This threshold was chosen based on previous works employing a similar technique.^12,14,41^ In controls, we found an average neuromelanin volume (111.9 mm^3^, SD of 33.49) similar to pathological estimates^41,43^ and to previous studies.^12,41^ Average aSN and pSN and overall average SN neuromelanin volumes were calculated.

To estimate LC neuromelanin signal, the three FSE T_1_-weighted images of each participant were co-registered and averaged, and a slice-to-slice intensity variation correction was applied. LC neuromelanin was estimated following the method described in Doppler *et al*.^44^ In brief, a ROI was drawn on a Montreal Neurological Institute (MNI) template following LC coordinates described in Keren *et al*.^45^ The contours of the ROIs were slightly enlarged to account for anatomical variation, and this generated a ‘search ROI’ that included the hyperintense signal of the LC without including other equally hyperintense structures. To investigate subregional LC differences, the search ROI was divided with two axial planes in three equal parts: rostral, middle and caudal. A background volume of interest was centred in the pons matching the extension of LC search ROIs (Supplementary Fig. 1D–F). Using SPM12, each subject’s FSE image was co-registered with their anatomical T_1_ image. The anatomical image was then spatially normalized using SPM’s segmentation tool. The LC ‘search ROIs’ in MNI space were then transformed to each subject’s FSE image using the inverse transformations. Then, a custom MATLAB (MathWorks, Natick, MS, USA) script employing SPM functions was used to extract and calculate the mean intensity of the five brightest connected voxels from the ‘search ROIs’ in each rostro-caudal subsection and from the background ROI. Neuromelanin-specific contrast was calculated as:


$$\mathrm{LC}\;\mathrm{neuromelanin}\;\mathrm{contrast}=\frac{\mathrm{mean}\;\mathrm{LC}\;\mathrm{ROI}\;\mathrm{intensity}\text{-}\mathrm{mean}\;\mathrm{background}\;\mathrm{ROI}\;\mathrm{intensity}}{\mathrm{mean}\;\mathrm{background}\;\mathrm{ROI}\;\mathrm{intensity}}.$$

The average (left and right) rostral, middle and caudal LC neuromelanin contrast was calculated.

### Dopamine transporter imaging

Dopamine transporter (DAT) single-photon emission computed tomography (SPECT) was performed at enrolment in iRBD patients alone.

Details of the DAT imaging procedure and acquisition are reported in the Supplementary material.

Specific binding ratios are calculated as:


$$\begin{array}{rl} & \mathrm{SBR}\;=\\ & \frac{\left(r\mathrm{egion}\;\mathrm{of}\;\mathrm{interest}\;\mathrm{count}\;\mathrm{density}\;\text{–}\;\mathrm{occipital}\;\mathrm{lobe}\;\mathrm{count}\;\mathrm{density}\right)}{\mathrm{Occipital}\;\mathrm{lobe}\;\mathrm{count}\;\mathrm{density}}. \end{array}$$

A *Z*-score, expressing the difference from the expected SBR (based on age and sex from the normal database), is also calculated and reported in SDs. A result below −2SDs is considered abnormal.

### Statistical analysis

Descriptive statistics were reported as means and SDs.

Group differences in SN and LC neuromelanin and in SN NODDI parameters (outcome variables) across HC, iRBD and PD were investigated with the following procedure: first, an omnibus ‘ANCOVA-type’ analysis was set up for each outcome variable, with group (HC, iRBD and PD) as a factor variable and age and sex as covariates. Significant omnibus tests (i.e. where a significant difference across groups was identified) were followed up with *post hoc* contrasts (six one-tailed paired comparisons representing the different combinations of group differences, i.e. HC > iRBD, HC < iRBD, etc.) investigating differences between each group; a family-wise error rate (FWER) Bonferroni correction was applied to *post hoc* tests *P*-values (corrected *P*-values are indicated as *P*_FWER_). This analysis was performed with Permutation Analysis for the Linear Model (PALM), an FSL tool designed to allow a ‘non-parametric’ permutation inference for the general linear model.^46^*P*-values of this analysis were calculated with the PALM permutation algorithm (with 10 000 permutations). Levene’s test was used to ensure homogeneity of variances of the outcome variables across groups.

Then, the associations between neuromelanin and diffusion parameters and between these and motor, cognitive and RBD scores were assessed through Pearson’s *r* correlation. Because the association analysis investigated mostly pathophysiological aspects of the disease (e.g. motor and SN neuroimaging abnormalities), which may be thought as continuous variables in the iRBD–PD spectrum, the associations were run in the combined PD-iRBD group. An exploratory analysis in the single subgroups is also provided in the Supplementary material. *P*-values of this group of tests were corrected using the false discovery rate (FDR) Benjamini–Hochberg procedure and are indicated with *P*_FDR_.^47^ These associations were performed using the average value of the neuromelanin signal extracted from left, right, anterior and posterior SN.

All statistical procedures, except for the PALM analysis, were carried out in Statistical Package for the Social Sciences version 26.0 (Armonk, NY, USA: IBM Corp.). For all statistical tests performed, the corrected *P*-value significance threshold was <0.05.

To provide an individual index of severity of MRI abnormalities, posterior SN FWF, posterior SN neuromelanin volume and middle LC neuromelanin contrast were converted to *w*-scores. Similar to *Z*-scores, *w*-scores are standardized scores but are corrected for covariates of interest. They have been used in neuroimaging studies to compare volumetric changes across control and disease cohorts^48,49^ and are calculated as follows:


$$w\;\mathrm{score}=\frac{\mathrm{observed}\;\mathrm{score}\;\mathrm{in}\;\mathrm{patient}\text{-}\mathrm{predicted}\;\mathrm{score}\;\mathrm{in}\;\mathrm{patient}}{\sqrt{\mathrm{residual}}\;\mathrm{variance}\;\mathrm{in}\;\mathrm{controls}},$$

where the predicted score in patient and residual variance in controls are estimated from a linear regression model conducted in controls with one outcome variable (pSN FWF, pSN neuromelanin or middle LC neuromelanin) and age and sex as covariates. This procedure transforms iRBD and PD scores in standard scores corrected for age and sex, thus providing the possibility of comparison with HC scores. Of note, *w*-scores in controls have a mean of 0 and a SD of 1; a *w*-score of 1.96 corresponds to the 2.5th percentile, 1.65 to the 5th percentile and 1.04 to the 15th percentile.

## Results

Table 1 shows descriptive demographic and clinical characteristics. Age (*H* = 0.638, *P* = 0.727) and sex (χ^2^ = 3.682, *P* = 0.159) were not significantly different across the iRBD, PD and HC groups.

**Table 1:** Demographics and clinical characteristics of the study cohort

	Healthy controls (HC)	iRBD	Parkinson’s disease	Statistical test, *P*-value
Number, *n*	18	14	18	
Sex, M/F	7/11	10/4	11/7	χ^2^ = 3.682, *P* = 0.159
Age, years	63.1 (11.0)	65.1 (7.9)	63.1 (9.0)	*H* = 0.638, *P* = 0.727
Disease duration (time since diagnosis), months		30.7 (36)	54.1 (70)	
Participants’ reported symptoms duration, months		74.45 (55)	75.82 (62)	
MoCA		25.9 (3.0)	27.3 (2.2)	U = 93.0, *P* = 0.220
RBDSQ	1.5 (1.4)	9.7 (1.8)	5.2 (3.9)	*H* = 27.007, *P* < 0.001*
MDS-UPDRS I		7.7 (4.9)	9.8 (6.1)	U = 92.0, *P* = 0.206
MDS-UPDRS II		2.0 (2.1)	8.4 (5.9)	U = 31.0, *P* < 0.001
MDS-UPDRS III		7.4 (5.9)	34.1 (14.6)	U = 6.0, *P* = <0.001

Scores are expressed as mean and SD, e.g. 10 (15).

χ^2^, chi-square statistic; F, female; H, Kruskal–Wallis test H statistic; iRBD, isolated REM sleep behaviour disorder; M, male; MDS-UPDRS, Movement Disorders Society—Unified PD Rating Scale; MoCA, Montreal Cognitive Assessment; *P*, *P*-value; RBDSQ, REM sleep behaviour disorders Screening Questionnaire; U, Mann–Whitney test statistic.

^*^HC-iRBD: standardized test statistics −5.19, *P*_FWER_ < 0.001; HC-PD: standardized test statistics −2.739, *P*_FWER_ = 0.018; PD-RBD: standardized test statistics 2.657, *P*_FWER_ = 0.024.

### Substantia nigra

iRBD participants did not show significant differences in SN neuromelanin signal or diffusion parameters compared with controls. PD participants showed significantly reduced pSN neuromelanin signal volume compared to both control and iRBD groups; no other parameter or contrast survived multiple comparison correction (Table 2 and Supplementary Table 1).

**Table 2:** Comparison between iRBD, PD and controls of diffusion and neuromelanin parameters in the SN and locus coeruleus. Detailed *post hoc* comparisons are also provided in Supplementary Table 1

Anatomical location	Group	value (mean, SD)	F (df = 45)	*P*-value	*Post hoc* test *P*-value^a^; FWER-corrected *α* level = 0.00833
Anterior SN NDI	HC	0.811 (0.084)	0.032	0.968	
	iRBD	0.841 (0.081)			
	PD	0.827 (0.061)			
Posterior SN NDI	HC	0.728 (0.089)	1.669	0.205	
	iRBD	0.734 (0.100)			
	PD	0.774 (0.104)			
Anterior SN FWF	HC	0.0837 (0.0660)	0.857	0.444	
	iRBD	0.0707 (0.0340)			
	PD	0.072 (0.0592)			
Posterior SN FWF	HC	0.0680 (0.0631)	1.648	0.203	
	iRBD	0.1022 (0.0796)			
	PD	0.1299 (0.1075)			
Anterior SN ODI	HC	0.261 (0.044)	1.372	0.372	
	iRBD	0.259 (0.044)			
	PD	0.277 (0.037)			
Posterior SN ODI	HC	0.259 (0.047)	1.136	0.335	
	iRBD	0.254 (0.039)			
	PD	0.273 (0.029)			
Anterior SN neuromelanin volume (mm^3^)	HC	83.03 (31.11)	1.948	0.159	
	iRBD	74.83 (28.93)			
	PD	61.89 (33.81)			
Posterior SN neuromelanin volume (mm^3^)	HC	28.87 (12.42)	5.386	0.007	PD versus HC: *P* = *0.0012* PD versus iRBD: *P* = *0.008*
	iRBD	25.41 (7.50)			
	PD	15.85 (13.14)			
Rostral LC neuromelanin contrast	HC	0.0751 (0.331)	1.564	0.219	
	iRBD	0.0534 (0.0398)			
	PD	0.0611 (0.0423)			
Middle LC neuromelanin contrast	HC	0.0873 (0.0273)	4.065	0.023	iRBD versus HC: *P* = *0.0065* PD versus HC: *P* = *0.0026*
	iRBD	0.0514 (0.0362)			
	PD	0.0540 (0.0446)			
Caudal LC neuromelanin contrast	HC	0.0331 (0.0368)	3.428	0.041	iRBD versus HC: *P* = 0.0134 PD versus HC: *P* = *0.0058*
	iRBD	−0.0051 (0.0513)			
	PD	−0.0012 (0.0459)			

*F*-statistics are computed from an ANCOVA model including age and sex. *Post hoc* comparisons are corrected for age and/or sex according to the significance of the covariates in the omnibus ANCOVA. Only relevant *post hoc* comparisons are reported.

df, degrees of freedom; *F*, *F*-statistic; FWER, family-wise error rate; FWF, free water fraction; HC, healthy controls; iRBD, isolated REM sleep behaviour disorder; LC, locus coeruleus; NDI, neurite density index; ODI, orientation dispersion index; PD, Parkinson’s disease; SN, substantia nigra.

^a^Italicized values indicate significance after Bonferroni’s correction (*α*/number of contrasts, where *α* = 0.05).

**Figure 1:**
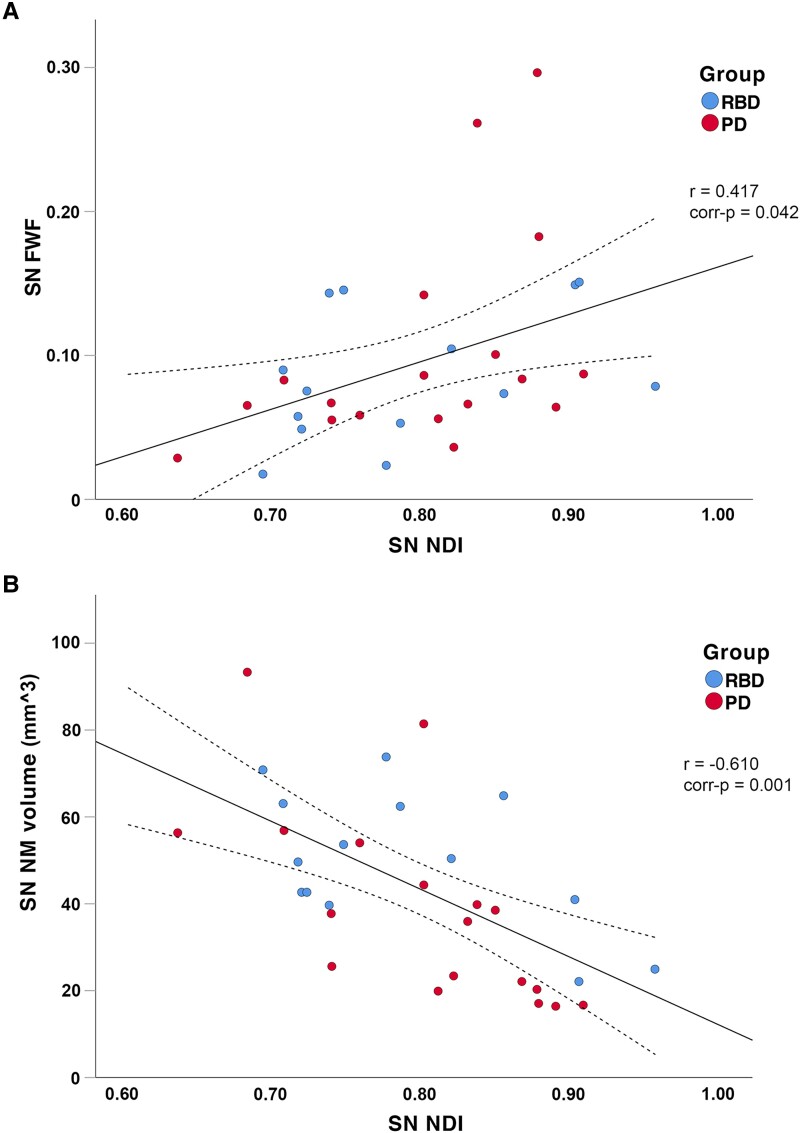
**Associations between NODDI and neuromelanin parameters in the SN.** Scatterplots showing the association between NDI and FWF (**A**, top panel) and between NDI and SN neuromelanin volume (**B**, bottom panel). The linear correlation line (continuous lines) and 95% confidence intervals (dotted lines) are shown. corr-*p*, FDR-corrected *P*-value; iRBD, isolated REM sleep behaviour disorder; mm^3^, cubic millimetres; PD, Parkinson’s disease; *r*, Pearson’s correlation coefficient.

In the combined iRBD–PD group, SN neuromelanin signal was associated with SN NDI (*r*_30_=−0.610, *P*_FDR_ = 0.001, Fig. 1) and SN ODI (*r*_30_=−0.486, *P*_FDR_ = 0.018); SN FWF was associated with SN NDI (*r*_30_ = 0.417, *P*_FDR_ = 0.042, Fig. 1). SN NDI was also associated with SN ODI (*r*_30_ = 0.494, *P*_FDR_ = 0.018). An exploratory association analysis was also run separately in PD and iRBD groups and is detailed in Supplementary Table 2.

DAT imaging was available in 13 of 14 iRBD participants; in these participants, average SN neuromelanin signal was associated with DAT availability in the most affected putamen (*r*_11_ = 0.665, *P*_FDR_ = 0.036), indicating that overall lower SN neuromelanin is associated with reduced DAT availability in the most affected putamen. No associations were found between putaminal DAT availability and diffusion parameters. Four iRBD participants had a DAT-SPECT *Z*-score < −2 SD of the normal mean in at least one striatal side, indicating a significant reduction in DAT availability compared with the corresponding age and sex group. One additional iRBD participant had a normal striatal *Z*-score but bilaterally reduced putaminal *Z*-score. At the last available clinical follow-up, two of these five subjects had phenoconverted to PD (both after an interval of 36 months since baseline DAT imaging). Another iRBD participant, with DAT availability within normal limits at enrolment, subsequently phenocoverted to PD.

MDS-UPDRS III score in the whole iRBD–PD group was associated with SN neuromelanin (*r*_30_=−0.404, *P*_FDR_ = 0.044) and with SN FWF (*r*_30_ = 0.486, *P*_FDR_ = 0.018). No associations were found with SN NDI and ODI. An exploratory association analysis was also run separately in PD and iRBD groups and is detailed in Supplementary Table 2.

### Locus coeruleus

**Figure 2:**
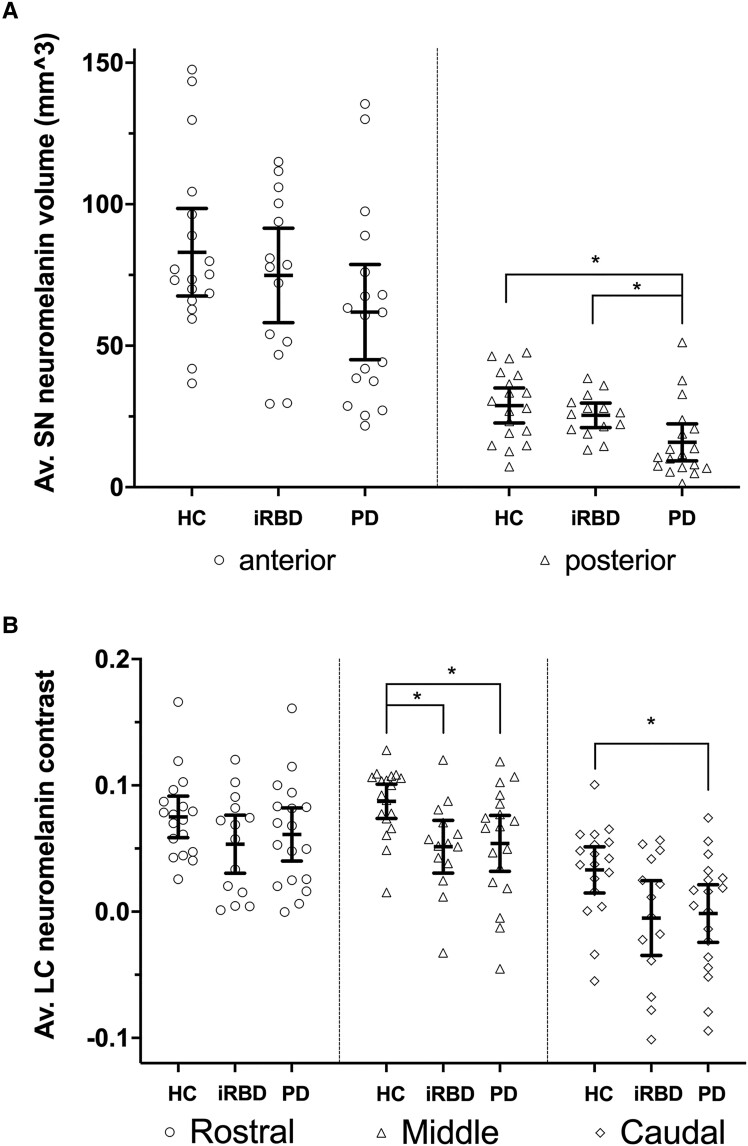
**SNand LC neuromelanin-associated signal.** Line plots showing mean and 95% confidence intervals for: (**A**) anterior and posterior SN average (Av.) volume and (**B**) LC rostral, middle and caudal average (Av.) neuromelanin contrast, in HC, iRBD and PD. Individual values are also shown. Asterisks indicate significant differences (after FWER correction) between single subgroups in *post hoc* tests that followed significant omnibus ANCOVA models. Specifically, for posterior SN neuromelanin volume (omnibus test *F* = 5.386, *P* = 0.007), PD versus HC: *T* = 3.018, *P*_FWER_ = 0.007, PD versus iRBD: *T* = 2.482, *P_FWER_* = 0.048; for middle LC neuromelanin contrast (omnibus test *F* = 4.065, *P* = 0.023), PD versus HC: *T* = 2.895, *P*_FWER_ = 0.016, iRBD versus HC: *T* = 2.643, *P*_FWER_ = 0.039; for caudal LC neuromelanin contrast (omnibus test *F* = 3.428, *P* = 0.041), PD versus HC: *T* = 2.562, *P*_FWER_ = 0.035. Detailed results are also reported in Table 2 and Supplementary Table 1. mm^3^, cubic millimetres.

A significant difference in neuromelanin-associated contrast was found in middle and caudal LC between iRBD, PD and HC groups (middle LC: *F*_45_ = 4.065, *P* = 0.023; caudal LC: *F*_45_ = 3.428, *P* = 0.041). No differences were seen in the rostral LC region. Compared with HC, both PD and iRBD participants showed significantly decreased middle LC neuromelanin contrast (iRBD: *T*_45_ = 2.643, *P*_FWER_ = 0.039; PD: *T*_45_ = 2.895, *P*_FWER_ = 0.016). In the caudal LC region, the PD group showed a significantly reduced neuromelanin contrast compared with HC (*T*_45_ = 2.562, *P*_FWER_ = 0.035), while that of iRBD did not survive multiple comparison correction (*T*_45_ = 2.314, *P*_FWER_ = 0.080). These results are also detailed in Fig. 2, Table 2 and Supplementary Table 1.

In the entire cohort of participants, reduced middle LC neuromelanin contrast was associated with higher RBDSQ scores (*r*_47_=−0.322, *P* = 0.024); no associations were found in the single subgroups. No associations with MoCA scores were found.

### Severity of MRI abnormalities

**Figure 3:**
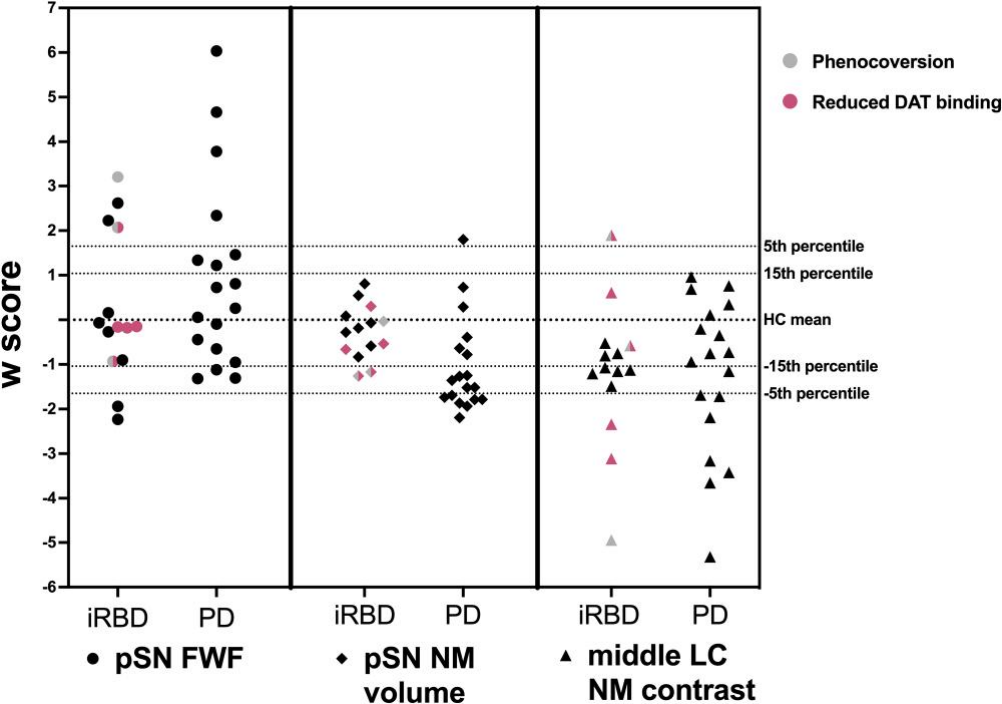
**Standardized *w*-scores for pSN FWF, pSN neuromelanin volume and middle LC neuromelanin contrast in iRBD and PD participants.** *w*-scores are standardized scores corrected for age and sex. They are calculated based on HC scores as detailed in the Statistical analysis section. In HC, the population mean is 0 and the SD is 1. A *w*-score of 1.65 corresponds to the 5th percentile and 1.04 to the 15th percentile. It is worth noting that higher FWF values represent greater abnormalities compared with controls, while lower neuromelanin values represent greater abnormalities compared with controls. The pink colour indicates iRBD participants with reduced putaminal dopamine transported binding (<−2 SD compared with the healthy population), and the grey colour indicates iRBD participants who phenoconverted. FWF, free water fraction; HC, healthy controls; iRBD, isolated REM sleep behaviour disorder; LC, locus coeruleus; NM, neuromelanin; PD, Parkinson’s disease; pSN, posterior substantia nigra.

To provide an individual participant’s index of MRI abnormalities severity in specific brain ROIs, *w*-scores corrected for age and sex were calculated for pSN FWF, pSN neuromelanin volume and middle LC neuromelanin. These are provided in Fig. 3.

## Discussion

In this study, a multimodal MRI investigation with neuromelanin-sensitive and dMRI was performed in iRBD and PD. In both iRBD and PD, a significant neuromelanin-associated signal reduction in the intermediate part of the LC was found. A similar reduction was also observed in the caudal part in PD. No significant differences were shown in SN neuromelanin signal or NODDI parameters in iRBD compared with controls. However, based on standardized and age- and sex-corrected *w*-scores, a few participants showed a degree of neuromelanin signal loss and FWF increase similar to that of PD participants. Furthermore, significant associations between reduced SN neuromelanin signal volume, NODDI parameters and motor impairment were shown.

Reduced middle LC neuromelanin signal coupled with normal SN parameters in the iRBD group may be indicative of the spatial heterogeneity of brain pathological involvement in this condition. iRBD is believed to show a body-first spread of pathological abnormalities, with initial gut involvement and overall ascending progression.^28,50^ Nonetheless, a more stochastic progression, for example based on structural connectivity, should also be considered.^51^ Therefore, the differential involvement of LC and SN could also be linked the different connections of these structures. A specific caudo-rostral topographical LC degeneration has previously been shown in PD, as opposed to a rostro-caudal gradient of pathology shown in Alzheimer’s disease.^44,52-54^ Doppler *et al*. found a neuromelanin signal loss that was greatest in the middle part of the LC in PD.^44^ With 7T-MRI, Madelung *et al.*^54^ showed a between-group (PD versus HC) difference in the middle and caudal sections, with greater differences towards the most caudal sections. A previous neuropathological study in PD observed degenerative changes in the entire LC, more prominently in the caudal and next in the middle segment.^55^ Based on this neuropathological observation, the authors also noted that degenerative process may begin in the middle segment and then spread towards the caudal segment of the LC as the disease advances. It should be acknowledged that in our study there was an overlap between some PD LC signal values and HC: previous studies have shown heterogeneity in the LC involvement in PD; therefore, not all PD patients show a significant involvement of LC.^13,56^ Conversely, a greater LC involvement may be related to the manifestation of RBD, which is usually accompanied by a more severe phenotype and degree of pathological involvement of brain structures.^57^ In iRBD, global LC neuromelanin loss has been shown previously, but evidence about subregional involvement was lacking.^6,7^ iRBD showed a similar degree and pattern of neuromelanin loss in the LC middle part compared with PD. This finding adds to the current literature that a selective involvement and degeneration pattern is evident in the LC middle part at the earliest stages of PD.

In the entire cohort, middle LC neuromelanin loss was associated with reported RBD severity. This finding may be indicative that middle LC degeneration is pathophysiologically linked to the generation of RBD symptoms.^7^ However, the severity of RBD symptoms may also be due to the degeneration of other brainstem structures, since REM sleep atonia is governed by a complex neural circuitry.^4,58^ LC neuromelanin loss was not associated with lower MoCA scores in PD and iRBD, a general screening measure of cognitive functioning. This finding may be due to multiple factors: dementia was an exclusion criterion in our study; the MoCA is a general cognitive function test; therefore, we were unable to investigate associations between LC degeneration and specific cognitive, e.g. attention deficits, where an association in PD has previously been shown^59^; the cohort was relatively small compared with previous studies where these associations were found.

SN neuromelanin-associated signal volume and NODDI parameters did not differ between iRBD and control groups. In PD, we found a significant reduction in pSN neuromelanin signal, while no significant increase in posterior SN FWF could be identified. Reduced pSN neuromelanin has been shown in several studies in PD, as well as an increase in free water.^60^ Regarding free water changes in pSN, several factors should be considered when interpreting the findings of this study: studies conducted in PD cohorts had greater numbers of participants^17,19,61,62^; very long TR during MRI acquisition may be more suitable to detect FW SN changes,^63,64^ although these may not be suitable for other dMRI analyses; free water estimates derived from single or multiple shells (i.e. NODDI used in the current study) acquisitions may yield similar yet not identical results.^62^ The latter issue should be further investigated in future studies to address similarities and differences.^16^

In iRBD, the finding of group-level SN neuromelanin-associated signal and NODDI parameters comparable to HC is not surprising, given that only five out of 13 participants had reduced striatal DAT availability and only two of these had converted to PD after an interval of 36 months since DAT imaging. Indeed, in the presence of early nigral degeneration, reduction of the DAT at the synaptic level may be an initial compensatory mechanism to maintain synaptic dopamine.^65^ Only a few studies in iRBD have shown increased free water^22,66^ and reduced SN neuromelanin^67^ when very sensitive volumetric estimates such as the one carried out in this study were used.^8^ It is difficult to compare our iRBD group to those in other studies in terms of motor severity, since one study^67^ used the older version of the UPDRS and another one did not report it.^22^ In one study,^68^ the mean MDS-UPDRS III in iRBD was higher than in our group (11.7 ± 6.5 versus 7.4 ± 5.9), likely underlying an overall closer proximity to motor phenoconversion. The relatively small number of iRBD participants could have also contributed to the lack of statistical significance in SN MRI parameters. However, several findings argue against this hypothesis, such as the significantly reduced neuromelanin signal elsewhere, i.e. in the LC, and the significantly higher posterior SN neuromelanin in the iRBD group compared with the PD group. Rather, these findings underlie the heterogeneity of this group. Indeed, it should be acknowledged that iRBD is a heterogeneous disorder in terms of disease progression;^3^ thus, it would be reasonable to expect a heterogeneous severity of neuroimaging abnormalities, especially in the SN, depending on the proximity to phenoconversion.

Interestingly, reduced SN neuromelanin signal volume was associated with raised NODDI parameters (NDI and ODI) in the entire disease group (PD and iRBD) and NDI was in turn associated with FWF. Free water, representing freely diffusing water molecules, is thought as a marker of neurodegeneration related to cell and axonal loss and inflammation.^24^ The NDI is a ratio between the intraneurite and intraneurite + extraneurite compartments,^30^ and it is thought to represent the amount of neurites (dendrites and axons) in a voxel.^29,31^ Practically, in normal tissue NDI is higher in white matter (because of the tight axons packing) and lower in grey matter (because of the more heterogeneous tissue organization, comprising dendrites and axons).^31^ Although the studies exploring its grey matter changes in neurodegeneration are scarce, it was suggested that its increase could be linked to cellular swelling and neuroinflammatory processes.^31,69,70^ In a previous study investigating PD and atypical parkinsonism against controls, PD, Multiple System Atrohy and Progressive Supranuclear Palsy did not show statistically significant differences in SN NDI compared with controls, yet mean values were higher coupled with higher FWF, a finding that is similar to our SN findings.^62^ The inverse association between SN neuromelanin signal volume and NDI could indicate that neuromelanin loss is accompanied by grey matter tissue remodelling. Furthermore, in iRBD SN neuromelanin signal loss was associated with reduced DAT availability in the most affected putamen. The association between different MRI techniques and across imaging modalities may reflect the ability of these techniques to capture different disease-related processes. Of note, no previous studies concomitantly explored SN free water and neuromelanin parameters in both PD and iRBD. Therefore, caution should be exerted when interpreting these finding because of the limited studies available *in vivo* and the lack of a specific pathological comparison of SN NODDI parameters. Future studies should investigate these associations longitudinally to ascertain whether they may be biomarkers of interest, e.g. in clinical trial settings.

Neuroimaging staging of disease progression in iRBD has been shown to be feasible by Knudsen *et al.*^6^ Investigating different neuroimaging aspects of neurodegeneration may be important to evaluate the effect of medication in future disease-modifying trials. Interestingly, posterior SN free water and neuromelanin signals showed lower degrees of abnormality in iRBD compared with PD at the group level, with only a few iRBD participants showing levels of abnormalities comparable to those in PD. While Knudsen *et al.* used a combination of molecular and MR imaging, we used a clinically feasible multimodal MRI protocol to investigate brain abnormalities in disease-specific locations and showed the possibility to capture disease-related neuroimaging abnormalities. Based on previous longitudinal studies with free water^19,22,63^ and neuromelanin-sensitive imaging,^9,16^ the use of MRI biomarkers to identify disease-related changes seems feasible and should be trialled when investigating disease-modifying agents.

This study has several limitations that should be addressed. The iRBD and PD subgroups are relatively small in sample size, which may have limited the statistical power of interrogations in the study, and this may have impacted some non-significant findings. Thus, the number of statistical tests was pragmatically kept to a minimum. It should also be pointed out that in the analysis of group means, it seemed appropriate to treat each subregion independently; therefore, *post hoc* comparisons were corrected for FWER for each subregion and not across subregions. We did not analyse side differences of neuroimaging parameters; rather, we used average left and right ROI values for analysis. While a detailed analysis on side differences was beyond the aims of the study, future studies should try to elucidate whether such side differences exist across different phenotypes of PD, especially for the LC, and whether they can be used as neuroimaging markers of a ‘disease-state’. DAT imaging was performed in iRBD participant only at enrolment, and this prevented comparison of their findings with those of PD participants. Anyway, DAT imaging in iRBD was compared to the manufacturer’s database of normal controls adjusted for age and sex to obtain *Z*-scores of differences. Due to the intrinsic characteristics of the neuromelanin-sensitive sequences acquired on the scanner used in this study, the analysis of the neuromelanin signal in LC and SN was carried out on two different sequences. While we recognize that from a clinical translation point of view it would be best to extract all data from one sequence, in this study we preferred optimizing the signal-to-noise ratio coming from the LC and SN ROIs. Microstructural analysis of the LC was limited to the neuromelanin signal. Although the LC offers a viable neuromelanin signal for analysis, diffusion analysis is currently limited by the small size of this nucleus, which is surrounded by other types of tissue (white matter bundles, CSF). These factors prevent the extraction of diffusion values from this structure at the field strength used in this study.

## Conclusion

In this study, we employed multimodal neuromelanin-sensitive and dMRI to investigate disease-related microstructural abnormalities in iRBD and PD. We showed that iRBD subjects have a similar pattern and degree of LC degeneration compared with PD, with the greatest neuromelanin-associated signal loss in the LC middle and caudal parts and an overall caudo-rostral pathological gradient. No significant differences were shown between iRBD and controls in the SN in this study. The associations between neuroimaging parameters acquired from different sequences, and with motor impairment, may indicate the possibility to capture different yet related tissue pathological characteristics. Overall, this study details MRI microstructural abnormalities in disease-related locations in iRBD and shows that a disease characterization through neuroimaging is feasible through MRI techniques that are becoming increasingly available. Longitudinal studies should complement these findings and their applicability should be tested in disease-modifying trials.

## Supplementary Material

fcaf023_Supplementary_Data

## Data Availability

Data used in the preparation of this manuscript are available upon request. The code generated in the analysis is available in the Supplementary material.
