# Supplementary material for: Substantia nigra and locus coeruleus microstructural abnormalities in isolated rapid eye movement sleep behaviour disorder and Parkinson’s disease
Source: Brain Commun. 2025 Jan 21;7(1):fcaf023. doi: 10.1093/braincomms/fcaf023 (PMC11806417; doi:10.1093/braincomms/fcaf023)
Supplement: fcaf023_Supplementary_Data [file fcaf023_supplementary_data.zip › Supplementary_material.docx]

**Supplementary material**

|  |  | T | p-value | Corrected α level | corrected p-value |
| --- | --- | --- | --- | --- | --- |
| Middle LC NM | HC > iRBD | 2.6438 | 0.0065 | 0.00833 | 0.039 |
|  | HC > PD | 2.8953 | 0.0026 | 0.00833 | 0.0156 |
|  | PD > iRBD | -0.0509 | 0.5246 | 0.00833 | 1 |
|  | iRBD > PD | 0.0509 | 0.481 | 0.00833 | 1 |
|  | iRBD > HC | -2.6438 | 0.9948 | 0.00833 | 1 |
|  | PD > HC | -2.8953 | 0.9965 | 0.00833 | 1 |

**Supplementary Table 1**. Post-hoc comparisons outputs from significant omnibus ANCOVA tests reported in Table 2.

|  |  | T | p-value | Corrected α level | corrected p-value |
| --- | --- | --- | --- | --- | --- |
| Caudal LC NM | HC > iRBD | 2.3141 | 0.0134 | 0.00833 | 0.0804 |
|  | HC > PD | 2.5624 | 0.0058 | 0.00833 | 0.0348 |
|  | PD > iRBD | -0.0709 | 0.5361 | 0.00833 | 1 |
|  | iRBD > PD | 0.0709 | 0.464 | 0.00833 | 1 |
|  | iRBD > HC | -2.3141 | 0.9878 | 0.00833 | 1 |
|  | PD > HC | -2.5624 | 0.9922 | 0.00833 | 1 |
|  |  | T | p-value | Corrected α level | corrected p |
| pSN NM | HC > iRBD | 0.3608 | 0.3596 | 0.00833 | 1 |
|  | HC > PD | 3.0175 | 0.0012 | 0.00833 | 0.0072 |
|  | PD > iRBD | -2.4815 | 0.9917 | 0.00833 | 1 |
|  | iRBD > PD | 2.4815 | 0.008 | 0.00833 | 0.048 |
|  | iRBD > HC | -0.3608 | 0.637 | 0.00833 | 1 |
|  | PD > HC | -3.0175 | 0.9972 | 0.00833 | 1 |

*Abbreviations*: HC: healthy controls; iRBD: isolated REM sleep behavior disorder; LC: locus coeruleus; NM: neuromelanin; PD: Parkinson’s Disease; SN: substantia nigra; T: T-statistic.

**Supplementary Table 2**. Exploratory association analysis between neuroimaging markers and motor scores run in iRBD and PD groups separately.

|  |  | **SN NM** | **SN NDI** | **SN FWF** | **SN ODI** |
| --- | --- | --- | --- | --- | --- |
| **iRBD (n = 14)** | **SN NM** | - |  |  |  |
|  | **SN NDI** | r=-0.540; p=0.046 | - |  |  |
|  | **SN FWF** | r=-0.597; p=0.024 | r=0.398; p=0.159 | - |  |
|  | **SN ODI** | r=-0.554; p=0.040 | r=0.667; p=0.008 | r=0.327; p=0.253 | - |
|  | **MDS-UPDRS III** | r=-0.302; p=0.294 | r=0.162; p=0.581 | r=0.471; p=0.089 | r=0.022; p=0.940 |
| **PD (n = 18)** | **SN NM** | - |  |  |  |
|  | **SN NDI** | r=-0.681; *p=0.002* | - |  |  |
|  | **SN FWF** | r=-0.132; p=0.601 | r=0.443; p=0.066 | - |  |
|  | **SN ODI** | r=-0.385; p=0.115 | r=0.303; p=0.221 | r=0.091; p=0.720 | - |
|  | **MDS-UPDRS III** | r=-0.317; p=0.200 | r=0.321; p=0.194 | r=0.646; *p=0.004* | r=-0.184; p=0.464 |

Reported p-values are uncorrected. Significant p-values after Benjamini-Hochberg correction are reported in *italic text*.

Abbreviations. FWF: free water fraction; iRBD: isolated REM sleep behavior disorder; MDS-UPDRS III: Movement Disorders Society – Unified Parkinson’s Disease Rating Scale; NDI: neurite density index; NM: neuromelanin; ODI: orientation dispersion index; PD: Parkinson’s Disease; SN: substantia nigra.

**Dopamine transporter imaging acquisition methods**

Patients were scanned 3–6h following a bolus intravenous injection of 185MBq of ^123^I-FP-CIT (Ioflupane (DaTSCAN) GE Healthcare, UK) (scan duration 25min) using a double-headed gamma camera (Siemens Symbia S or Siemens Intevo) fitted with a low-energy high-resolution parallel hole collimator. A total of 120 (60 per detector) 25s views over a 360° orbit were acquired on a 128×128 matrix with a zoom of 1.23× giving a pixel size 3.9×3.9mm. Images were on reconstructed using iterative reconstruction with resolution recovery, uniform attenuation correction and Monte Carlo scatter correction. For all images, transverse data was manually reoriented to correct for any head tilt and to provide a consistent display. Reconstructed images were registered to a normal template in 3D space and volume of interest of the caudate, putamen and occipital lobes were applied bilaterally to the registered scan in BRASS v5.1.1 (Hermes Medical Solutions, Stockholm, Sweden).

**Data availability: code generated and used in this work**

**1. FSL (FMRIB Software Library) PALM (Permutation Analysis for the Linear Model)^1^ code was run in GNU Octave 6.2.0^2^ as a standalone software running on a UNIX machine.**

1. The omnibus ANCOVA F-test was run with the following command:

‘palm -i *ROI*.csv -d master_matrix.mat -t omnibus_contrasts.con -f omnibus_f_test.fts -o *ROI/* -savemetrics -savedof’

Where:

- “-i *ROI*.csv” is the comma separated value table including the ROI values for all groups
- “-d master_matrix.mat” is the design matrix including a factor with three groups (HC, iRBD, PD); age and sex as mean-centered covariates.
- “-t omnibus_contrasts.con” is the contrasts table specifying the T-contrasts (group, age, sex)
- “-f Omnibus_f_test.fts” is the table specifying the F-test for the factor variable “group”
- “-o *ROI/*” is the output folder

1. The post-hoc test was run with following command:

‘palm -i *ROI*.csv -d master_matrix.mat -t post_hoc.con -o *ROI_post_hoc/* -corrcon -savemetrics -savedof’

Where:

- “-i *ROI*.csv” is the comma separated value table including the ROI values for all groups
- “-d master_matrix.mat” is the design matrix including a factor with three groups (HC, iRBD, PD); age and/or sex (according to the significance of the covariates in the omnibus model) as mean-centered, nuisance covariates.
- “-t post_hoc.con” is the contrasts table specifying the T-contrasts (HC>PD, HC>iRBD, PD>iRBD, PD>HC, iRBD>HC)
- “-o *ROI/*” is the output folder
- “-corrcon” is the command to perform FWER p-value correction across the specified contrasts.

**2. Code used in SPM12 running in MATLAB® for slice-to-slice intensity variation correction**

function mjf_slice_intensity(varargin)

%mjf 2021 08 to use an image and mask, gets the mean intensity within the mask for the odd and even

%slices, and then scales the odd slices so the overall intensity matches the even ones.

%possibly could add an option to ignore (or otherwise cope without a mask).

%version 0.1

p=inputParser;

p.addParameter('fname','noimage', @ischar);

p.addParameter('fmask','noimage', @ischar);

p.addParameter('dout','noimage', @ischar);

p.addParameter('endslices',0,@(x)any(x==[0 1],2)); %also adjust top and bottom slices

p.parse( varargin{:});

inp = p.Results;

%spm get files.

if strcmp(inp.fname,'noimage')

fns=spm_select(1,'image','Select image file');

else

fns=inp.fname;

end

if strcmp(inp.fmask,'noimage')

fnmask=spm_select(1,'image','Select mask file');

else

fnmask=inp.fmask;

end

if strcmp(inp.dout,'noimage')

outdir=spm_select(1,'dir','Select output directory');

else

outdir=inp.dout;

end

comma1=strfind(fns,',1');

if (comma1),

inptim=fns(1:comma1-1);

else

inptim=fns;

end

comma1=strfind(fnmask,',1');

if (comma1),

inptmask=fnmask(1:comma1-1);

else

inptmask=fnmask;

end

[pathmask,filemask,ext]= fileparts(inptmask);

mask_vol = spm_vol(fullfile(pathmask,[filemask ext]));

mask_im = spm_read_vols(mask_vol);

[pathim,fileim,ext]= fileparts(inptim);

im_vol = spm_vol(fullfile(pathim,[fileim ext]));

im_im = spm_read_vols(im_vol);

if inp.endslices

mask_middle = mask_im;

mask_middle(:,:,end)=0;

mask_middle(:,:,1)=0;

mask_ends = mask_im;

mask_ends(:,:,2:end-1) = 0;

end

% remove first and last slices (which may well have different intensity due to excitation profile).

mask_im(:,:,end)=0;

mask_im(:,:,1)=0;

%get odd and even slice masks.

mask_odd = mask_im;

mask_even = mask_im;

mask_odd(:,:,2:2:end)=0;

mask_even(:,:,1:2:end)=0;

odd_val = mean(im_im(mask_odd > 0));

even_val = mean(im_im(mask_even > 0));

%and rescale the odd slices

odd_ratio = odd_val/even_val;

im_im(:,:,2:2:end) = im_im(:,:,2:2:end)*odd_ratio;

if inp.endslices

mid_val = mean(im_im(mask_middle>0));

end_val = mean(im_im(mask_ends>0));

im_im(:,:,1) = im_im(:,:,1)*mid_val/end_val;

im_im(:,:,end) = im_im(:,:,end)*mid_val/end_val;

end

%write out with sc prefix.

VO= im_vol;

VO.fname = fullfile(outdir,['sc' fileim ext]);

spm_write_vol(VO,im_im);

**3. Code used in SPM12 running in MATLAB® to find the brigthest voxels inside a locus coeruleus predefined region of interest.**

function mjf_findbright(varargin)

%mjf 2021 08 to find mean of brightest N voxels, and brightest N 'connected voxels'

% //TODO check images are single volume and load.

% am asssuming that the images are integer.

%version 0.1

p=inputParser;

p.addParameter('fname','noimage', @ischar);

p.addParameter('fmask','noimage', @ischar);

p.addParameter('dout','noimage', @ischar);

p.addParameter('nbright',10,@(x)x>0 && x<600);

p.addParameter('nconnect',12,@(x)x>0 && x<600);

p.addParameter('saveconnect',1,@(x)any(x==[0 1],2));

p.parse( varargin{:});

inp = p.Results;

%spm get files.

if strcmp(inp.fname,'noimage')

fns=spm_select(1,'image','Select image file');

else

fns=inp.fname;

end

if strcmp(inp.fmask,'noimage')

fnmask=spm_select(1,'image','Select mask file');

else

fnmask=inp.fmask;

end

if strcmp(inp.dout,'noimage')

outdir=spm_select(1,'dir','Select output directory');

else

outdir=inp.dout;

end

comma1=strfind(fnmask,',1');

if (comma1),

inptmask=fnmask(1:comma1-1);

else

inptmask=fnmask;

end

[pathmask,filemask,ext]= fileparts(inptmask);

mask_vol = spm_vol(fullfile(pathmask,[filemask ext]));

mask_im = spm_read_vols(mask_vol);

mask_values = unique(mask_im);

% remove the ',1' indicatint that we are looking at volume 1, other

% wise spm_write_vol complains ...

disp(fns(1,:));

comma1=strfind(fns(1,:),',1');

if (comma1),

inpt=fns(1,1:comma1-1);

else

inpt=fns(1,:);

end

[pathname,filename,ext]= fileparts(inpt);

% optionally we are saving the connected masks.

% so create a blank to put them in

if inp.saveconnect

inp_vol = spm_vol(fullfile(pathname,[filename ext]));

inp_im = spm_read_vols(inp_vol);

connectmask = inp_im*0;

connectmaskname=fullfile(outdir,['connect' num2str(inp.nconnect) '_' filename '_' filemask ext]);

end

% go through each separate mask label in the mask file

brightest = cell(length(mask_values),1); %output

for mm = 1:length(mask_values)

mval = mask_values(mm);

brightest{mm}.mval=mval;

brightest{mm}.ind=NaN;

brightest{mm}.conn=NaN;

if mval < 0.1 || isnan(mval)

%background so ignore

continue

end

%apply the mask to the image, store in temporary file

tmpout=fullfile(pathmask,[filemask 'tmp12345mjf' ext]);

formul = sprintf('i1.*(i2>(%d-0.5)).*(i2<(%d+0.5))',mval,mval);

spm_imcalc({inpt, inptmask},tmpout,formul);

%read in the temporary masked file

inp_vol = spm_vol(tmpout);

inp_im = spm_read_vols(inp_vol);

%resize to a vector and sort

inp_voxels = inp_vol.dim(1)*inp_vol.dim(2)*inp_vol.dim(3);

rs = sort(reshape(inp_im, inp_voxels,1));

%and find the brightest

brightest{mm}.ind = mean(rs((inp_voxels-inp.nbright+1):end));

%to get the brightest n connected, start with the intensity of the nth bright pixel

% and decrease until we find something

thr = inp_im*0;

init_bright = rs(inp_voxels-inp.nconnect+1);

for tt = init_bright:-1:1

thr(inp_im >= tt) =1;

CC = bwconncomp(thr,26);

brightlist={};

for jj=1:length(CC.PixelIdxList)

% if this blob has exactly the correct number, then add to list.

% if it has too many, remove least bright least connected pixels and

% then add.

if length(CC.PixelIdxList{jj}) == inp.nconnect

brightlist{end+1}=CC.PixelIdxList{jj};

elseif length(CC.PixelIdxList{jj}) > inp.nconnect

tmpplist = CC.PixelIdxList{jj};

while length(tmpplist) >inp.nconnect

%get pixels in sorted order

[~,sorder] = sort(inp_im(tmpplist));

okflag=false;

%remove each pixel in turn, and see if it is still a N connected thing.

for s=1:length(sorder)

tmpplist2 = tmpplist(sorder([1:(s-1) (s+1):end]));

thr2 = thr*0;

thr2(tmpplist2)=1;

CC2 = bwconncomp(thr2,26);

if length(CC2.PixelIdxList{1}) >=inp.nconnect

tmpplist = tmpplist2;

okflag=true;

break;

end

end

end

if okflag

brightlist{end+1}=tmpplist;

end

end

end

%did we find anything?

if ~isempty(brightlist)

%if so find the brightest of them

nbright=-1;

maxbright=-1;

for jj=1:length(brightlist)

tmpbright = mean(inp_im(brightlist{jj}));

if tmpbright > maxbright

maxbright = tmpbright;

nbright = jj;

end

end

brightest{mm}.conn = maxbright;

% if save connected voxels, label the output

if inp.saveconnect

connectmask(brightlist{nbright}) = mval;

end

break

end

end

end

%results

outfile = fullfile(outdir,'findbright.csv')

fid=-1;

headerst='filename,maskname,roi_val,nbright,mean_bright,nconnected,mean_connected\n';

fprintf(headerst)

if ~exist(outfile,'file')

fid=fopen(outfile,'w');

fprintf(fid,headerst);

else

fid = fopen(outfile,'a');

end

%output mean values to screen and file

for mm = 1: length(mask_values)

if brightest{mm}.mval > 0 && ~isnan(brightest{mm}.mval)

outfileall=sprintf('%s,%s,',fullfile(pathname,[filename ext]), ...

fullfile(pathmask,[filemask ext]));

outfilenam=sprintf('%s,%s,',[filename ext], ...

[filemask ext]);

outs = sprintf('%d,%d,%g,%d,%g\n',...

round(brightest{mm}.mval),...

inp.nbright,brightest{mm}.ind,...

inp.nconnect,brightest{mm}.conn);

fprintf('%s',[outfilenam outs]); %to screen

fprintf(fid,'%s',[outfileall outs]); %to file

end

end

fclose(fid);

%optionally save connected mask.

if inp.saveconnect

VO= inp_vol;

VO.dt = [2 0]; %unsigned Char

VO.pinfo(1)=1; %set intensity scaling to 1.

VO.fname = connectmaskname;

spm_write_vol(VO,connectmask);

end

%remove temporary file

delete(fullfile(pathmask,[filemask 'tmp12345mjf' ext]));

**Supplementary References**

1. Winkler AM, Ridgway GR, Webster MA, Smith SM, Nichols TE. Permutation inference for the general linear model. Neuroimage. 2014;92(100):381-397. doi:10.1016/j.neuroimage.2014.01.060
2. John W. Eaton, David Bateman, Søren Hauberg, Rik Wehbring (2020). GNU Octave version 6.1.0 manual: a high-level interactive language for numerical computations. URL: https://www.gnu.org/software/octave/doc/v6.1.0/
