# Supplementary figures and images for: Substantia nigra and locus coeruleus microstructural abnormalities in isolated rapid eye movement sleep behaviour disorder and Parkinson’s disease
Source: Brain Commun. 2025 Jan 21;7(1):fcaf023. doi: 10.1093/braincomms/fcaf023 (PMC11806417; doi:10.1093/braincomms/fcaf023)

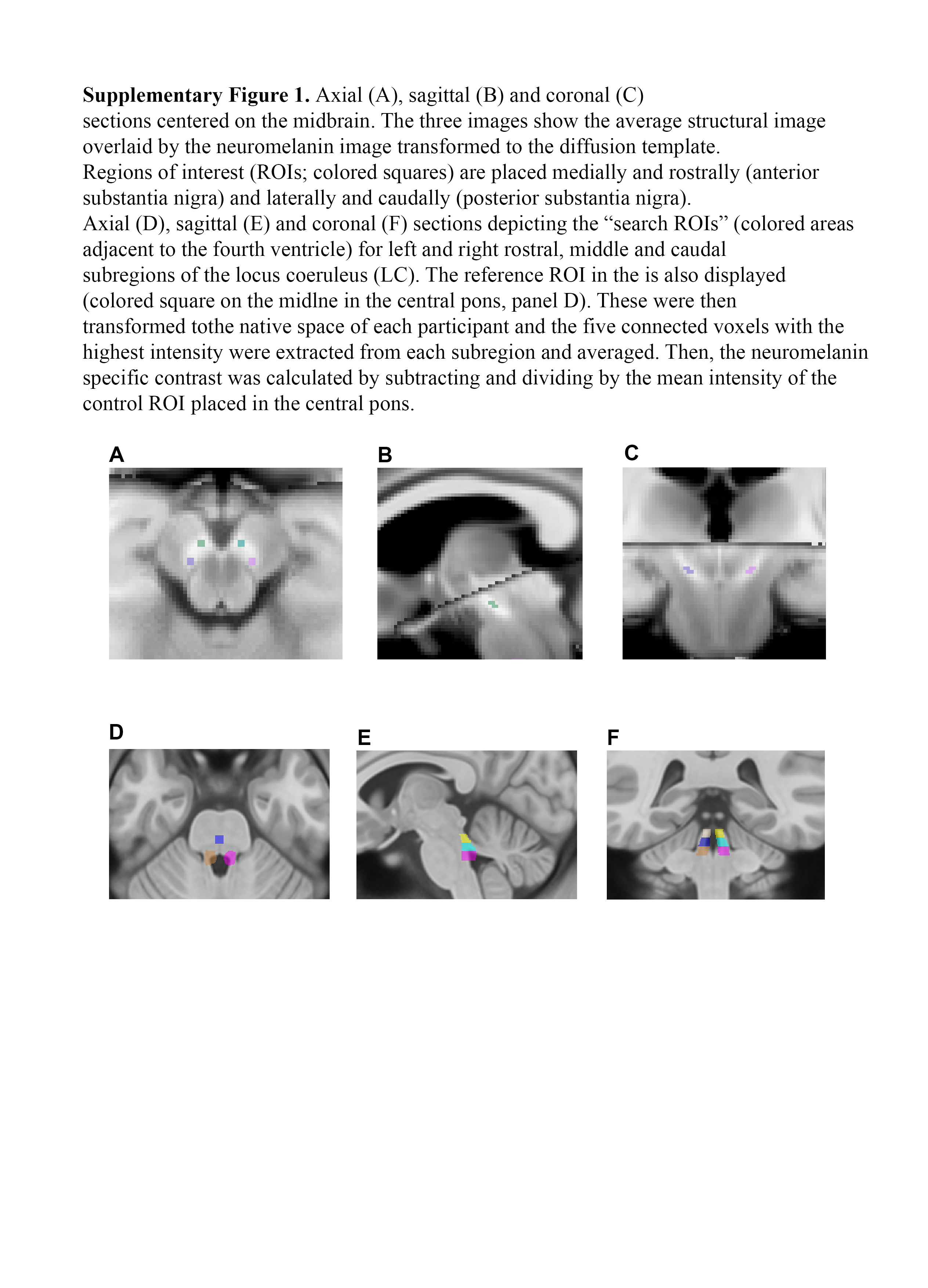

Supplement: fcaf023_Supplementary_Data [file fcaf023_supplementary_data.zip › Supplementary_Figure.tif]
